# Beneficial effect of polyphenols in COVID‐19 and the ectopic F_1_F_O_‐ATP synthase: Is there a link?

**DOI:** 10.1002/jcb.30306

**Published:** 2022-07-15

**Authors:** Isabella Panfoli, Alfonso Esposito

**Affiliations:** ^1^ Dipartimento di Farmacia (DIFAR) Università di Genova Genoa Italy; ^2^ Computational Biology Unit International Centre for Genetic Engineering and Biotechnology, ICGEB Trieste Italy

**Keywords:** COVID‐19, F1Fo‐ATP synthase, polyphenols

## Abstract

COVID‐19 has been proposed to be an endothelial disease, as endothelial damage and oxidative stress contribute to its systemic inflammatory and thrombotic events. Polyphenols, natural antioxidant compounds appear as promising agents to prevent and treat COVID‐19. Polyphenols bind and inhibit the F_1_F_o_‐ATP synthase rotary catalysis. An early target of polyphenols may be the ectopic F_1_F_o_‐ATP synthase expressed on the endothelial plasma membrane. Among the pleiotropic beneficial action of polyphenols in COVID‐19, modulation of the ecto‐F_1_F_o_‐ATP synthase, lowering the oxidative stress produced by the electron transfer chain coupled to it, would not be negligible.

The novel β‐coronavirus SARS‐CoV‐2 emerged in December 2019 was recognized as a pandemic on March 11, 2020, by the World Health Organization (WHO).^1^ As of May 6, 2022, 513 955 910 confirmed cases of COVID‐19, including 6 249 700 deaths, of COVID‐19 have been reported worldwide.^2^ The availability of effective vaccines based on different platforms worldwide has changed the COVID‐19 scenery,^3^ although there is concern about novel variants and waning of protection over time.^4^

Systemic inflammation, endothelial damage, and abnormal coagulation are the hallmarks of the novel coronavirus infectious disease COVID‐19.^5^ COVID‐19 is an acute respiratory disease; however, many severe cases develop life‐threatening multiorgan dysfunction that may not transition from the pulmonary infection.^6^ COVID‐19 is associated with an increased risk of arterial and venous thromboembolic events in critically ill patients.^7^, ^8^ The endothelial cell (EC) expressing the angiotensin‐converting enzyme type 2 (ACE2), is a target of SARS‐CoV‐2.^9^ Consistently, COVID‐19 has been proposed to be an endothelial disease,^10^ where a vascular inflammation would promote oxidative stress and thrombus formation.^11^ It has previously been proposed that an early EC dysfunction in COVID‐19 may induce a pro‐oxidant status.^12^ The expression of a functional F_1_F_o_‐ATP synthase (i.e., coupled to an electron transfer chain, ETC) on the surface of ECs was reported.^13^, ^14^, ^15^, ^16^ It was supposed that in COVID‐19, the virus would damage the EC plasma membrane, as well as the proteins therein expressed. An impairment of the ETC ectopically residing on the EC membrane would produce reactive oxygen species (ROS), in turn priming the EC to acquire a pro‐inflammatory and prothrombotic phenotype.^12^ In fact, the ETC is a major ROS producer.^17^

The F_1_F_o_‐ATP synthase (ATP synthase, or Complex V) is the nanomotor that produces the bulk of cell ATP in the presence of a proton gradient generated by the ETC, by a rotary mechanism.^18^ It is expressed not only on the inner mitochondrial membrane but also in ectopic locations,^19^ among which neuronal surface,^20^ photoreceptor outer segment,^21^ and cell plasma membranes.^22^, ^23^, ^24^ 

Polyphenols are a large group of bioactive natural phytochemicals, divided into multiple subclasses,^25^ known for their antioxidant, anti‐inflammatory, and immunomodulatory properties.^26^, ^27^ Notably, it was demonstrated by X‐ray crystallography that polyphenols such as resveratrol, quercetin, and piceatannol bind the mitochondrial F_1_F_o_‐ATP synthase, specifically targeting a hydrophobic pocket between the gamma and beta subunits of its F_1_ catalytic domain, as shown in Figure 1 inhibiting its rotary catalysis,^28^ consistently with previous biochemical data.^29^ Table in Figure 1 reports the IC50 values for six polyphenols assayed by three different studies.^30^, ^31^, ^32^

**Figure 1:**
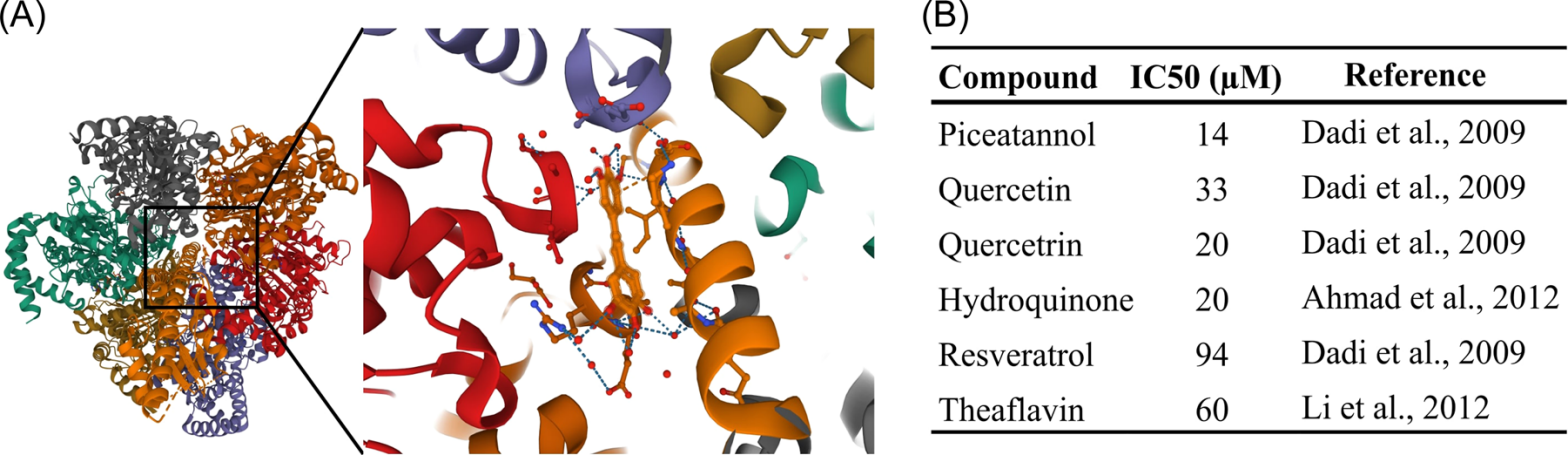
Crystal structure of the bovine mitochondrial F_1_F_o_‐ATP synthetase inhibited by resveratrol as solved by X‐ray crystallography by Gledhill et al.,^28^ retrieved searching the term 2JIZ on the PDB database. In the zoom on the binding site, the interactions between the macromolecule and the ligand are visible as dashed lines, blue dashed lines are hydrogen bonds, whereas the orange dashed line is a cation‐pi interaction between the resveratrol and Lys260 on the Gamma chain (A). Table showing the IC50 for six polyphenols assayed by three different studies for their inhibitory effects on ATP synthase (B).

Several papers reported that polyphenols inhibit the catalytic activity of the F_1_F_o_‐ATP synthase.^28^, ^31^, ^32^, ^33^ In a model of ecto‐F_1_F_o_‐ATP synthase expression, inhibition by polyphenols lowered the ROS production by the ETC coupled to it.^34^, ^35^ The inhibition of the EC ecto‐F_1_F_o_‐ATP synthase by angiostatin was proven to bear antiangiogenic effects.^16^

Evidence supports the potential applicability of polyphenols in the prevention and treatment of COVID‐19,^36^ due to their antioxidant, anti‐inflammatory, and potential antiviral properties.^37^, ^38^, ^39^, ^40^, ^41^ Moreover, some polyphenols, have been recently approved in clinical trials for COVID‐19 prevention and/or therapy.^41^, ^42^ Quercetin has been extensively studied for the treatment of COVID‐19 patients.^43^, ^44^, ^45^, ^46^, ^47^, ^48^ An overlap between resveratrol targets and SARS‐CoV‐2 differentially expressed genes was demonstrated.^49^ The use of green tea polyphenols in the management of COVID‐19 has been also proposed.^50^ On the other hand, it has been observed that current COVID‐19 treatments can potentially cause nutrition‐drug interactions, negatively affecting nutritional status also by acting on the intestinal microbiota, in turn partly responsible for the metabolism of polyphenols in turn affecting their availability.^51^

Even though polyphenols are antioxidants, and their scavenging ability can directly lower ROS levels, modulation of the F_1_F_o_‐ATP‐synthase rotary catalysis by polyphenols may not be negligible when considering the overall beneficial action of these natural compounds in COVID‐19. It is tempting to suppose that polyphenols could modulate the ecto‐F_1_F_o_‐ATP synthase expressed onto the EC plasma‐membrane in case of dysfunction due to the SARS‐CoV‐2 binding to it. This, in turn, would lower the ROS production by the imbalanced ecto‐ETC being coupled to the F_1_F_o_‐ATP synthase. Evidence indicates that ROS damage plays a critical role in COVID‐19.^52^ This would be one of the pleiotropic positive actions^53^ polyphenols exert on COVID‐19, and notably the earliest, as it would occur in the blood, where bioavailability is optimal. Since the ETC is a major producer of ROS, modulating the F_1_F_o_‐ATP synthase would hamper the vascular luminal oxidative stress, the ultimate trigger of the inflammation and thrombus formation in COVID‐19. Notably, the inhibition of the ecto‐ F_1_F_o_‐ATP synthase would also lower the concentration of the extracellular ATP, thus limiting the activation of the P2 purinergic receptors, among which P2X7, key mediators of the vast array of biological effects, among which the pro‐inflammatory and pro‐thrombotic ones.^54^ The hypothesis presented here, may help in expanding the mechanism by which polyphenols can modulate SARS‐CoV‐2 pathogenic actions.

## CONFLICT OF INTEREST

The authors declare no conflict of interest.
